# Positioning Diverse Type IV Structures and Functions Within Class 1 CRISPR-Cas Systems

**DOI:** 10.3389/fmicb.2021.671522

**Published:** 2021-05-21

**Authors:** Hannah N. Taylor, Eric Laderman, Matt Armbrust, Thomson Hallmark, Dylan Keiser, Joseph Bondy-Denomy, Ryan N. Jackson

**Affiliations:** ^1^Department of Chemistry and Biochemistry, Utah State University, Logan, UT, United States; ^2^Department of Microbiology and Immunology, University of California, San Francisco, San Francisco, CA, United States

**Keywords:** CRISPR, Cas, type IV, Cas7, Cas6, DinG helicase, CysH

## Abstract

Type IV CRISPR systems encode CRISPR associated (Cas)-like proteins that combine with small RNAs to form multi-subunit ribonucleoprotein complexes. However, the lack of Cas nucleases, integrases, and other genetic features commonly observed in most CRISPR systems has made it difficult to predict type IV mechanisms of action and biological function. Here we summarize recent bioinformatic and experimental advancements that collectively provide the first glimpses into the function of specific type IV subtypes. We also provide a bioinformatic and structural analysis of type IV-specific proteins within the context of multi-subunit (class 1) CRISPR systems, informing future studies aimed at elucidating the function of these cryptic systems.

## Introduction

Clustered Regularly Interspaced Short Palindromic Repeats-CRISPR associated (CRISPR-Cas) prokaryotic defense systems utilize Cas1 and Cas2 proteins, along with system-specific proteins such as Cas4, IHF, Csn2, and Cas9, to integrate foreign genetic material into the CRISPR locus, immunizing the cell against viruses and plasmids (Datsenko et al., 2012; Yosef et al., 2012; Nuñez et al., 2014, 2016; Heler et al., 2015; Rollie et al., 2015; Wang et al., 2015; Wei et al., 2015; Sternberg et al., 2016; Jackson S. A. et al., 2017; Kieper et al., 2018; Lee et al., 2018). To provide immunity, the CRISPR locus is transcribed and processed by RNA nucleases into CRISPR derived RNAs (crRNAs) (Brouns et al., 2008; Marraffini and Sontheimer, 2008; Haurwitz et al., 2010; Deltcheva et al., 2011). The crRNAs combine with Cas proteins to form ribonucleoprotein (RNP) complexes, which recognize and bind complementary nucleic acids. Binding induces cleavage of the foreign nucleic acid, protecting the cell (Brouns et al., 2008; Carte et al., 2008; Hale et al., 2008, 2009; Marraffini and Sontheimer, 2008; Garneau et al., 2010; Jackson R. N. et al., 2017; Hille et al., 2018).

Although all CRISPR systems use these general mechanisms to achieve immunity, the systems themselves are remarkably diverse, comprising two classes (1–2), six types (I–VI), and at least 33 subtypes (Yan et al., 2019; Makarova et al., 2020). In class 2 systems (types II, V, VI) a single Cas protein binds the crRNA to form the RNP complex, while class 1 RNP complexes (types I, III, IV) bind the crRNA with several proteins. Of the six CRISPR-Cas types, the least understood is type IV. Recent bioinformatic, biochemical, and structural studies of type IV CRISPR-Cas systems have provided valuable insights into type IV system function. Here we compile known data on type IV systems, highlight recent advances in type IV system biology and biochemistry, and indicate questions concerning type IV systems that need to be addressed. Additionally, we provide phylogenetic analyses that suggest ancillary proteins associated with type IV systems have evolved Cas-specific functions.

## Type IV Systems are Minimal, Mobile CRISPR-Cas Systems

Distinguishable from other CRISPR-Cas systems, Type IV systems encode a distinct *cas7*-like gene (*csf2)*, lack adaptation genes, rarely encode an obvious nuclease, and are primarily found on plasmids (Koonin and Makarova, 2017, 2019; Pinilla-Redondo et al., 2019). These unique features of type IV systems have made it difficult to predict the function of type IV systems.

**FIGURE 1:**
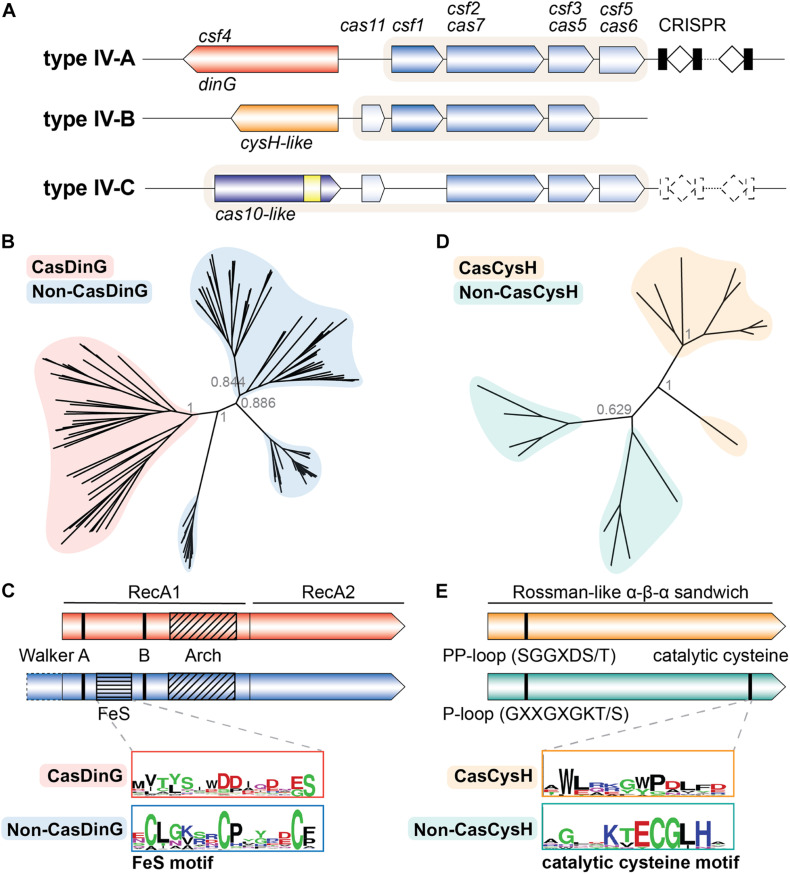
The type IV Cas accessory proteins have evolved a Cas specific function. **(A)** Classification schematic of type IV CRISPR-Cas systems. A typical locus is represented for each type IV subtype. Dashed lines indicate components that are sometimes not encoded by the subtype. Shaded backgrounds highlight which gene products form the ribonucleoprotein (RNP) complex. The yellow square in the IV-C *cas10-like* large subunit represents an HD nuclease domain. **(B)** Phylogenetic tree of Cas- and non-CasDinG sequences. Posterior probabilities are shown. **(C)** Cartoons of Cas- and non-CasDinG sequences indicating positions of certain helicase motifs and domain architecture. Weblogos (Crooks, 2004) of the FeS cluster region in non-CasDinG (below, blue outline) and CasDinG (top, red outline) are shown. **(D)** Phylogenetic tree of Cas- and non-CasCysH sequences. Posterior probabilities are shown. **(E)** Cartoons of Cas- and non-CasCysH sequences. CasCysH is predicted to adopt the Rossman-like α - β - α fold observed in non-CasCysH structures. Positions and sequences of P- and PP-loops are indicated. Weblogos of the catalytic cysteine in non-CasCysH (bottom, teal outline) and CasCysH (top, orange outline) are shown.

All type IV systems encode homologs of proteins known to form multi-subunit RNP complexes, explaining their class 1 designation. However, the presence of specific genes, gene arrangements, and differences in gene sequences have been used to further classify type IV systems into three distinct subtypes (IV-A, IV-B, and IV-C) (Makarova et al., 2011, 2015, 2020). Types IV-A, IV-B, and IV-C each contain a subtype-specific gene (*dinG, cysH-like*, and *cas10-like*, respectively) and subtype-specific features (Figure 1A). Type IV-A operons encode the three core type IV genes (*csf1, csf2*, and *csf3*), an endoribonuclease (*cas6*/*csf5*), a CRISPR array, and a putative helicase (*dinG*). Type IV-B operons encode the three core type IV genes and a *cas11-like* gene but lack a CRISPR locus. Additionally, type IV-B operons contain an ancillary gene, labeled *cysH-like* because the predicted secondary structure of its protein product resembles the CysH enzyme (Shmakov et al., 2018; Faure et al., 2019). Type IV-C systems encode *csf2* and *csf3*, but in place of the *csf1* gene they encode a *cas10-like* gene with a putative HD-nuclease domain. They also encode the *cas11-like* gene observed in IV-B systems, and sometimes a *cas6* RNA endonuclease and CRISPR array (Pinilla-Redondo et al., 2019; Makarova et al., 2020).

It is curious that the type IV systems that encode CRISPR loci do so in the absence of adaptation genes. It has been hypothesized that these type IV systems commandeer adaptation machinery from other CRISPR-Cas types to maintain their CRISPRs, similar to some type III systems (Staals et al., 2013, 2014; Elmore et al., 2015; Bernheim et al., 2020). Supporting this hypothesis, recent bioinformatic work showed that some type IV-A subtypes co-localize with certain type I systems (e.g., I-F, I-E), and that spacers found within co-localized type IV CRISPR loci appeared to be selected with the same criteria utilized by the type I system adaptation machinery [e.g., both I-E and IV-A protospacers are flanked with an 5′-AAG-3′ protospacer adjacent motif (PAM)] suggesting there may be functional cross-talk between these systems (Pinilla-Redondo et al., 2019). To confirm this proposed cooperation, *in vivo* and *in vitro* experimental work that examines adaptation in type IV systems with adaptation proteins from co-localized systems is needed.

## The *cas7*-Like Gene, *csf2*, Distinguishes Type IV From Other Class 1 Systems

Initial bioinformatic analyses proposed *csf1* as the type IV *cas* signature gene (Makarova et al., 2015). However, some type IV systems lacking *csf1* have been identified, necessitating that the type IV *cas7* homolog, *csf2*, be used to classify type IV systems (Pinilla-Redondo et al., 2019). In type I and type III systems, Cas7-like proteins bind the crRNA guide within a helical backbone of a multi-subunit RNP complex and make direct interactions with other protein subunits (Jore et al., 2011; Lintner et al., 2011; Staals et al., 2013; Jackson et al., 2014; Mulepati et al., 2014; Osawa et al., 2015). Similarly, a recent cryo-EM structure of the type IV-B RNP complex revealed that Csf2 proteins bind RNA within a helical backbone, indicating a conserved function for Cas7-like proteins in all class 1 systems (Zhou et al., 2021). Despite this conservation, the sequence and structure of Csf2 is distinguishable from other Cas7 proteins (Makarova et al., 2011; Supplementary Figure 1). For example, when representative Cas7 sequences from all class 1 subtypes were aligned and a phylogenetic tree created, Csf2 sequences clustered on a separate branch from type I and type III Cas7 sequences (Supplementary Figure 2A and Supplementary Methods). Csf2 is distinct from other Cas7 homologs but appears to be most closely related to type III, supporting evolutionary hypotheses that type IV systems diverged from type III systems (Koonin and Makarova, 2019; Pinilla-Redondo et al., 2019; Makarova et al., 2020). Interestingly, an alignment of only Csf2 sequences shows clustering of Csf2 from each type IV subtype on its own branch, illustrating the intrinsic diversity of type IV subtypes and suggesting subtype-specific functional distinctions (Supplementary Figure 2B). It is worth noting that the type IV-B Csf2 subunit structure is most similar to the structure of the Cas7 homolog in type III-A systems, Csm3 (Zhou et al., 2021). Csm3 contains a catalytic aspartate that cleaves RNA targets (Tamulaitis et al., 2014). Alignment of target-bound Csm3 with Csf2 indicates that, although Csf2 also contains a conserved aspartate residue in a similar location, it is not in a position amenable for target cleavage (Zhou et al., 2021). Additional structural studies of type IV complexes bound to nucleic acid targets and complementary biochemical assays are needed to determine whether Csf2 is capable of RNA nuclease activity.

## Type IV-A Systems are Defense Systems With an Unknown Mechanism of Action Involving a DinG Helicase

Recently, a type IV-A system from *Pseudomonas aeruginosa* was shown to exhibit crRNA-guided defense against plasmids (Crowley et al., 2019), consistent with an analysis of type IV CRISPR spacers that suggested type IV-A systems disproportionately target plasmids (Pinilla-Redondo et al., 2019). Notably, earlier bioinformatic work indicated that many type IV-A spacers target viruses and prophage sequences encoding putative anti-CRISPRs, suggesting type IV-A systems also actively target viruses (Shmakov et al., 2017; Yin et al., 2019; Nobrega et al., 2020). However, direct data, such as viral plaque assays, are needed to confirm that type IV-A systems protect against viral attack.

Structural and biochemical work on a type IV-A complex from *Aromatoleum aromaticum* and IV-A Cas6 from *Mahella australiensis* demonstrated that the RNA endonuclease Csf5/Cas6 processes a crRNA upon which Csf1, Csf2, Csf3, and Csf5 form an RNP complex (Özcan et al., 2018; Taylor et al., 2019). At least three distinct crRNA processing endoribonucleases are encoded by Type IV-A systems (Cas6, Csf5, and Cas6e) (Makarova et al., 2020; Supplementary Figure 3A). Sequence alignments between biochemically characterized and putative type IV Csf5/Cas6 enzymes revealed Csf5 enzymes cleave RNA with arginine active site residues, while type IV Cas6 and Cas6e enzymes utilize histidine/tyrosine active site residues (Supplementary Figures 3B, 4). Despite these obvious differences in endoribonucleases, we hypothesize that in all type IV-A systems the Csf1, Csf2, Csf3, and Csf5/Cas6 proteins bind to the processed crRNA to form a multi-subunit complex that binds complementary nucleic acid.

It remains unclear whether type IV RNP complexes bind single stranded RNA [like the type III Csm and Cmr complexes (Hale et al., 2009; Samai et al., 2015)] or double stranded DNA [like the type I Cascade complexes (Brouns et al., 2008)] and how type IV complexes distinguish self from non-self targets. RNPs that target dsDNA usually rely on a protein-mediated binding event with a specific non-self sequence adjacent to the complementary target, called a PAM (protospacer adjacent motif) (Mojica et al., 2009; Westra et al., 2012, 2013). PAM binding provides the energy for target duplex unwinding and interrogation of the DNA by the crRNA-guide. Work by Pinilla-Redondo et al. (2019) identified a consensus PAM (5′-AAG-3′) flanking protospacers targeted by a subset of type IV-A systems, suggesting type IV-A systems rely on PAM recognition to license binding. However, the consensus PAM may only reflect a preference of the acquisition machinery, which may explain why consensus PAM sequences have not been identified in all IV-A systems. Reliance on a specific PAM sequence for type IV-A RNP interference remains to be confirmed experimentally, but it should be noted that a promiscuous PAM recognition mechanism may indicate that the type IV complexes have evolved to accommodate the preferences of diverse Cas1 and Cas2 proteins that use different PAM sequences in spacer acquisition.

Interestingly, the structural similarities of the type IV-B complex to the type III Csm complex suggest that type IV complexes may target RNA (Zhou et al., 2021). Instead of recognizing a “non-self” PAM to license base pairing with a double-stranded DNA target, RNPs that bind RNA generally use a “self recognition” mechanism to distinguish self from non-self sequences (Marraffini and Sontheimer, 2010). Self-sequence located in the flanking regions of a bound RNA can base pair with the direct repeat of the crRNA disrupting downstream activation of effector nucleases (You et al., 2019). Self-sequences are inhibitory to overall immune function (Marraffini and Sontheimer, 2010; Elmore et al., 2016; Estrella et al., 2016; Kazlauskiene et al., 2016; Zhang et al., 2016; Han et al., 2017; Liu et al., 2017), but in some systems only a subset of non-self protospacer flanking sequences [called RNA-PAMs (rPAM) in type III systems or protospacer flanking sites (PFS) in type VI systems] are activating (Marraffini and Sontheimer, 2010; Abudayyeh et al., 2016; Elmore et al., 2016). We suspect that one or more Csf subunits may be responsible for PAM recognition to license DNA binding or rPAM recognition to activate immunity. We anticipate that *in vivo* PAM screens and biochemical binding assays with purified type IV-A RNPs will reveal the type IV-A self vs. non-self recognition mechanism.

Type IV-mediated plasmid clearance required all type IV-A system genes (*csf1, csf2, csf3, csf5*, and *dinG/csf4*) and a CRISPR containing a spacer complementary to a target plasmid sequence adjacent to a 5′-TTC-3′ PAM (Crowley et al., 2019). Because deleting the *dinG* gene or mutating the ATPase active site residues (DEAH-box) fully disrupted plasmid clearance, we hypothesize that RNP complex binding recruits the type IV-associated DinG (CasDinG) helicase to the bound target and CasDinG-mediated ATP binding and hydrolysis performs work, such as duplex unwinding, that is essential for plasmid clearance. Such a mechanism is similar to the more extensively studied type I Cas3 helicase-nuclease that unwinds and degrades dsDNA targets bound by the type I Cascade RNP complex (Beloglazova et al., 2011; Mulepati and Bailey, 2011; Sinkunas et al., 2011).

Both DinG and Cas3 classify as superfamily 2 helicases but, unlike Cas3, CasDinG proteins have no identifiable nuclease domain and have yet to be biochemically or structurally characterized (Fairman-Williams et al., 2010; Makarova et al., 2020). DinG helicases are generally involved in DNA recombination and repair, and are classified by amino acid sequence motifs involved in ATP binding and hydrolysis and nucleic acid binding and translocation (Lewis et al., 1992; Voloshin et al., 2003; Voloshin and Camerini-Otero, 2007; McRobbie et al., 2012; Wu and Brosh, 2012; Thakur et al., 2014; Cheng and Wigley, 2018). The motifs are located across two RecA helicase domains (Supplementary Figure 5). The first helicase domain also harbors two insertions, an iron sulfur cluster domain, and an arch domain, which are both important for duplex strand splitting (Ren et al., 2009; Peissert et al., 2020).

Since non-CasDinG helicases and their homologs have been extensively studied biochemically and structurally, we hypothesized that an in-depth comparison of CasDinG with non-CasDinG sequences would provide insight to CasDinG function. To investigate the relationship of CasDinG to other DinG helicases, we compiled CasDinG and non-CasDinG sequences from organisms containing a type IV-A system and generated a phylogenetic tree (Supplementary Methods; Figure 1B). Interestingly, CasDinG and non-CasDinG sequences clustered separately even when the sequences were retrieved from the same organism, suggesting CasDinG is functionally distinct from non-CasDinG. Notably, CasDinG helicases contain insertions within the first RecA domain of the same lengths as the iron-sulfur and arch insertions, but they lack homology with non-CasDinG sequences, including the residues important for coordinating the iron-sulfur cluster (Figure 1C). Sequence differences in these regions suggest these inserts may be a source of functional distinctions important for defense activities. Many functions for CasDinG have been hypothesized, including a role in displacing bound RNP complexes, cleaving bound targets with an unidentified nuclease activity (perhaps housed within an insert), or recruitment of endogenous nucleases to bound targets (Grodick et al., 2014). Notably, DinG helicases have been observed in a few type I and III systems (Dwarakanath et al., 2015; Makarova et al., 2020), indicating an evolutionary link and suggesting that some CasDinG activities essential for type IV immunity may have been co-opted by other class 1 systems.

In summary, recent bioinformatic and *in vivo* studies have indicated type IV-A systems protect prokaryotes from plasmids and viruses, but the mechanisms that underpin how the Csf RNP complex and CasDinG work together to provide immunity remain to be determined.

## Type IV-B Systems Encode an RNP Complex of Unknown Function and a Specialized cysH-Like Protein With Putative ATP α-Hydrolase Activity

Unlike type IV-A and IV-C subtypes, type IV-B systems lack a CRISPR locus and a crRNA processing enzyme, and are associated with an ancillary gene identified as *cysH-like* by the HHpred secondary structure prediction and alignment tool (Zimmermann et al., 2018; Makarova et al., 2020; Figure 1A). A recent structural study recombinantly expressed and purified a *Mycobacterium sp. JS623* IV-B Csf RNP complex containing four type IV-B proteins (Csf1, Csf2, Csf3, and Cas11) (Zhou et al., 2021). Interestingly, RNA sequencing revealed the type IV-B Csf complex bound small heterogeneous RNAs, instead of co-expressed type I-E crRNAs from the *Mycobacterium sp. JS623* plasmid, suggesting a possible function other than CRISPR-mediated defense. A high resolution cryo-EM structure of the complex revealed several Csf2 subunits bind an RNA within a helical filament, while Cas11 subunits form a minor filament that contacts the larger filament at Csf2 dimer interfaces (Zhou et al., 2021). This structure of intertwined large and small protein filaments is similar to other class 1 RNP complexes, suggesting similar function as an RNA-guided complex that binds complementary targets (Jore et al., 2011; Lintner et al., 2011; Staals et al., 2013; Jackson et al., 2014; Mulepati et al., 2014; Osawa et al., 2015; Supplementary Figure 6).

Several observations are currently confounding an understanding of the type IV-B complex function. First, electron density for Csf1 and Csf3 subunits was not clearly observed within the structure, although SDS-PAGE indicated their presence in the purified complex. Thus, the structure and function of these important proteins remains unknown. Second, because the IV-B Csf complex bound heterogenous RNA, it remains unknown whether the Csf complex lacks sequence-specific preference for small RNAs or if the RNA(s) that the complex would normally bind were not available in the recombinant expression conditions. Finally, the role of the strictly conserved ancillary CysH-like protein and how it may interact with the complex is unknown.

The key to understanding the function of type IV-B systems likely lies with the uncharacterized, but ubiquitous, type IV-B accessory *cysH*-like gene (Shmakov et al., 2018; Faure et al., 2019). Typical CysH proteins are phosphoadenosine phosphosulfate (PAPS) reductases involved in sulfate assimilation. Structures reveal CysH proteins fit within a family of enzymes that adopt a Rossman-like α–β–α sandwich fold that binds nucleotides (InterPro IPR014729) (Blum et al., 2020). CysH proteins also contain a P-loop motif (GXXGXGKT/S consensus sequence) that binds nucleotide phosphates, and a conserved C-terminal cysteine that performs nucleophilic attack on the PAPS β-sulfate, hydrolyzing PAPS at the α-phosphate and forming a covalent thiosulfanate intermediate during sulfur reduction (Savage et al., 1997; Carroll et al., 2005; Chartron et al., 2007). Interestingly, the DndC protein from the recently discovered DND bacterial immune system also belongs to the PAPS reductase family, and uses a similar mechanism to incorporate sulfur into the backbone of chromosomal DNA through a disulfide cysteine (You et al., 2007; Wang et al., 2018; Faure et al., 2019). These phosphorothioate modifications serve as an epigenetic signature that allows the DND system to distinguish self from non-self DNA (Wang et al., 2018). The predicted structural homology between the type IV-B CysH (CasCysH) and DndC proteins justifies speculation that CasCysH proteins perform a similar function. However, a closer analysis of type IV-B CasCysH sequences suggests that if CasCysH does epigenetically modify DNA, it will not be through the formation of phosphorothioates. Although HHPred predicts CasCysH adopts a Rossman-like α–β–α sandwich fold, the catalytic cysteine important for sulfonate reduction in non-CasCysH and phosphothiolation of DNA by DndC is absent. Additionally, the P-loop sequence of CasCysH is more similar to the PP-motif (pyrophosphatase motif) (SGGXDS/T consensus sequence) observed in ATP PPases (Bork and Koonin, 1994; Figure 1E).

To better understand CasCysH activity and to explore the relationship between non-CasCysH and CasCysH proteins, sequences from organisms encoding both Cas- and non-CasCysH were aligned and phylogenetic trees determined. As was seen with Cas- and non-CasDinG, CasCysH sequences cluster separately from non-CasCysH sequences even when the sequences were retrieved from the same organisms (Figure 1D). Together, with our more in depth sequence analysis these differences suggest CasCysH evolved to preserve nucleotide binding without sulfonucleotide reduction.

Non-CasCysH enzymes fall within the larger classification of ATP α-hydrolases, which include N-type ATP PPases (Savage et al., 1997). Unlike non-CasCysH and DndC, N-type ATP PPases catalyze sequential reactions involving substrate AMPylation, instead of the formation of covalent enzyme substrate intermediates requiring nucleophilic attack from a catalytic cysteine (Chartron et al., 2007; Wang et al., 2018). The absence of a catalytic cysteine suggests that the role of CasCysH is to stabilize the AMPylation of specific substrates through catalysis of ATP α-hydrolase activity. We hypothesize that such an activity could be used to modify nucleic acids bound by the type IV-B RNP for immune system purposes, gene regulation, or the formation of secondary messengers. Future biochemical studies aimed at defining the function of CasCysH and its interactions with the IV-B Csf RNP complex will be critical for understanding type IV-B systems.

Several hypotheses exist concerning the function of type IV-B CRISPR-Cas systems. As they lack both a CRISPR array and an obvious nuclease it seems unlikely that type IV-B systems function as independent CRISPR-Cas defense systems (Makarova et al., 2011; Faure et al., 2019; Zhou et al., 2021). It has been suggested that type IV-B systems could bind crRNAs derived from other CRISPR systems, forming IV-B RNP complexes that perform RNA-guided defense (Makarova et al., 2011, 2015; Koonin and Makarova, 2019). As type IV systems are generally encoded on plasmids, such a crRNA scavenging system could be passed between organisms, acting as a mobile defense system. Interestingly, it was recently shown that sometimes type IV-B systems colocalize with specific class 1 systems, suggesting a cooperative function (Pinilla-Redondo et al., 2019). However, the same study showed that type IV-B systems are most often observed without other CRISPR systems, supporting an alternative hypothesis that proposes IV-B systems may protect plasmids from RNA-guided defense mechanisms by sponging up and inactivating small guide RNAs, including crRNAs (Koonin and Makarova, 2017; Faure et al., 2019; Pinilla-Redondo et al., 2019). Such an anti-guide-RNA activity could give plasmids containing a type IV-B system a selective advantage (Shmakov et al., 2018; Koonin and Makarova, 2019). Although intriguing, neither of these hypotheses explain the role of the highly conserved ancillary protein CasCysH, suggesting the true function of IV-B systems may be more intricate than has so far been proposed.

## The Newly Classified Type IV-C System Highlights the Diverse Nature of Type IV CRISPR-Cas Systems

Only recently did bioinformatics studies classify the subtype IV-C CRISPR-Cas system (Pinilla-Redondo et al., 2019; Makarova et al., 2020). Type IV-C systems lack a Csf1 subunit, and instead encode a Cas10-like subunit with an HD nuclease domain (Figure 1A). Type III CRISPR-Cas systems also encode Cas10, which is the large subunit of the RNP complex. In type III systems Cas10 has nuclease activity and synthesizes cyclic oligoadenylate second messengers (Jung et al., 2015; Kazlauskiene et al., 2017; Niewoehner et al., 2017). The type IV Cas10 contains an HD nuclease domain but not a nucleotide cyclase motif “GGDD,” suggesting it has nuclease but not cyclic adenylate synthetase activity (Pinilla-Redondo et al., 2019). Interestingly, the HD domain motifs of type IV Cas10 are more similar to the HD domain of Cas3 than the type III Cas10 (Aravind and Koonin, 1998; Makarova et al., 2020). The presence of a *cas10-like* gene in type IV-C systems and the similarity between the type III-A and type IV-B RNP complexes support proposals that type IV and type III CRISPR-Cas systems share a common ancestor (Pinilla-Redondo et al., 2019; Makarova et al., 2020; Zhou et al., 2021). Experimental work is needed to better understand the function of these fascinating systems.

Several variants of type IV systems have been identified in bioinformatics studies and clinical samples which include type IV systems; without a *csf1*, with a *csf1-csf3* fusion, with a *recD* helicase instead of *dinG*, and in association with IncH1b plasmids (Crowley et al., 2019; Pinilla-Redondo et al., 2019; Kamruzzaman and Iredell, 2020; Newire et al., 2020). We expect further study and analysis of these diverse systems will reveal unique mechanisms and functions.

## Unanswered Questions Concerning Type IV Biochemistry and Biological Function

**FIGURE 2:**
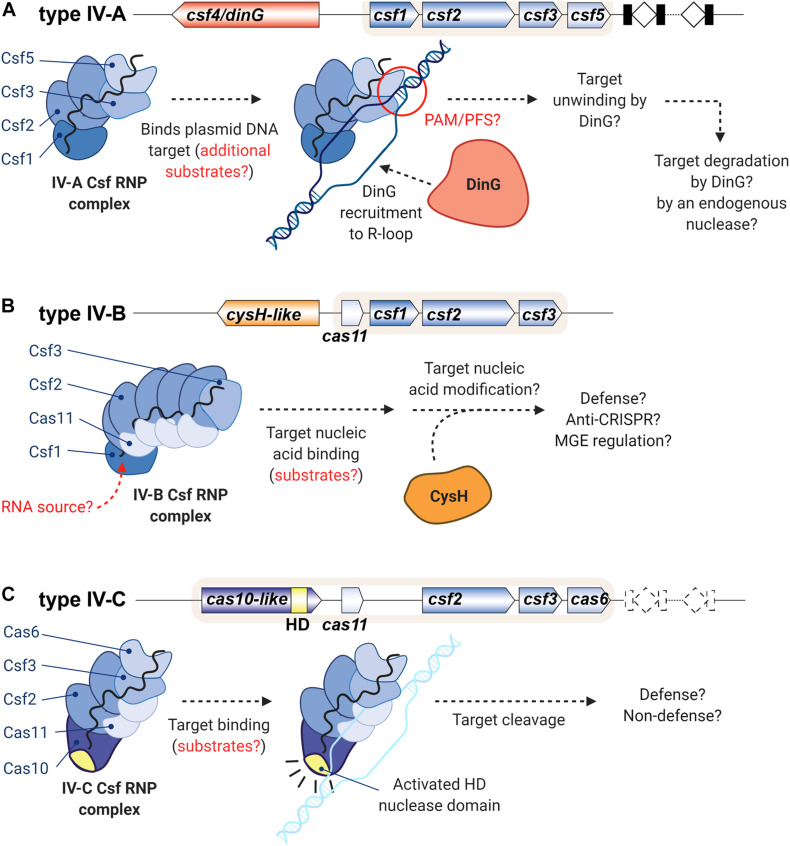
Models of type IV system functions highlighting questions that remain to be answered. **(A)** IV-A RNP complexes likely bind DNA targets and recruit CasDinG for target unwinding and degradation. **(B)** IV-B RNP complexes likely interact with CasCysH to perform an unknown function. **(C)** The putative IV-C RNP complex likely binds a nucleic acid target and cleaves that target with the HD nuclease domain. Created with BioRender.com.

Throughout this perspective we have highlighted pressing questions concerning type IV CRISPR-Cas system structures and functions. Here we suggest models for the function of each type IV subtype and indicate areas which require further understanding. Type IV-A systems have been shown to form RNP complexes and prevent targeted plasmid transformation, but they have not been shown to target viruses nor is the mechanism of crRNA-guided defense clear (Figure 2A). Understanding the targets of the type IV-A system is critical to understanding the full scope of its defense activity. The presence of a helicase within the system suggests the need to unwind a duplex substrate. We suspect that the type IV-A system targets dsDNA, as it can defend against invasive plasmids (Crowley et al., 2019). However, CasDinG could also be important for unwinding duplex secondary structure within ssRNA targets or for targeting dsRNA phages (Poranen and Tuma, 2004). Remaining questions include the targeting parameters of the complex (DNA vs RNA, seed sequence, mismatch tolerance), how the complex distinguishes self from non-self, the role of CasDinG in immunity, and how targets are cleared without an identifiable nuclease domain within the system. We speculate that the IV-A RNP complex will bind to a dsDNA target and recruit CasDinG to the resulting R-loop, allowing CasDinG to unwind the target. To clear the target from the cell, we hypothesize that either an endogenous nuclease will degrade the unwound nucleic acid, or CasDinG harbors an intrinsic nuclease activity not predicted by the sequence.

The function of type IV-B Csf RNP complexes is still unknown (Figure 2B). Many questions of type IV-B system function will be answered as the source of the RNA component of the IV-B RNP complex is discovered and the function of the accessory protein CasCysH is understood. We propose that the Csf RNP complex will bind a nucleic acid target and recruit CasCysH to modify the nucleic acid via an ATP α-hydrolase activity.

No biochemical studies have been performed with type IV-C systems, to date. We hypothesize that the IV-C Csf proteins will form an RNP complex with a crRNA and the Cas10-like subunit (Figure 2C). The IV-C Csf RNP complex will bind a nucleic acid target complementary to the crRNA and the HD nuclease domain of the Cas10-like subunit will cleave the target. Some IV-C systems have a CRISPR and a crRNA processing endonuclease and others do not, suggesting some IV-C systems may serve a crRNA-guided defense function while others may employ Cas proteins to perform an entirely different, non-defense function. Future studies should seek to understand the role of Cas10 within the type IV Csf RNP complex and the overall function of type IV-C CRISPR-Cas systems.

To understand the function of type IV CRISPR-Cas systems, it is critical that we determine the structures and biochemical functions of the type IV subtype specific proteins: CasDinG, CasCysH, and Cas10-like. Phylogenetic trees suggest that the IV-A DinG and IV-B CysH have evolved to support a putative Cas specific function. The IV-C Cas10 also has a unique domain composition that likely supports a distinct function. We propose that, due to the different accessory proteins and subtype specific proteins encoded by the three subtypes, each type IV subtype will have a distinct mechanism of action and possibly distinct function. We highly anticipate future work detailing the mechanisms and functions of type IV RNP complexes and their accessory proteins.

## Data Availability Statement

The original contributions presented in the study are included in the article/Supplementary Material, further inquiries can be directed to the corresponding author.

## Author Contributions

HT, TH, and RJ conceived and wrote the manuscript. HT, EL, MA, TH, and DK performed alignments and generated phylogenetic trees. All authors provided critical feedback on the manuscript.

## Conflict of Interest

JB-D is a scientific advisory board member of SNIPR Biome and Excision Biotherapeutics, and a scientific advisory board member and co-founder of Acrigen Biosciences. The remaining authors declare that the research was conducted in the absence of any commercial or financial relationships that could be construed as a potential conflict of interest.
