# Supplementary material for: Positioning Diverse Type IV Structures and Functions Within Class 1 CRISPR-Cas Systems
Source: Front Microbiol. 2021 May 21;12:671522. doi: 10.3389/fmicb.2021.671522 (PMC8175902; doi:10.3389/fmicb.2021.671522)
Supplement: Supplementary file 1 [file Data_Sheet_1.docx]

Supplementary Material

# Supplementary Data

**Supplementary Data File 1.** Compiled sequences for Cas- and non-CasDinG, and Cas- and non-CasCysH,

**Supplementary Data File 2.** Selected sequences used for Cas7 and Cas6 alignments and phylogenetic trees.

# Supplementary Figures

**
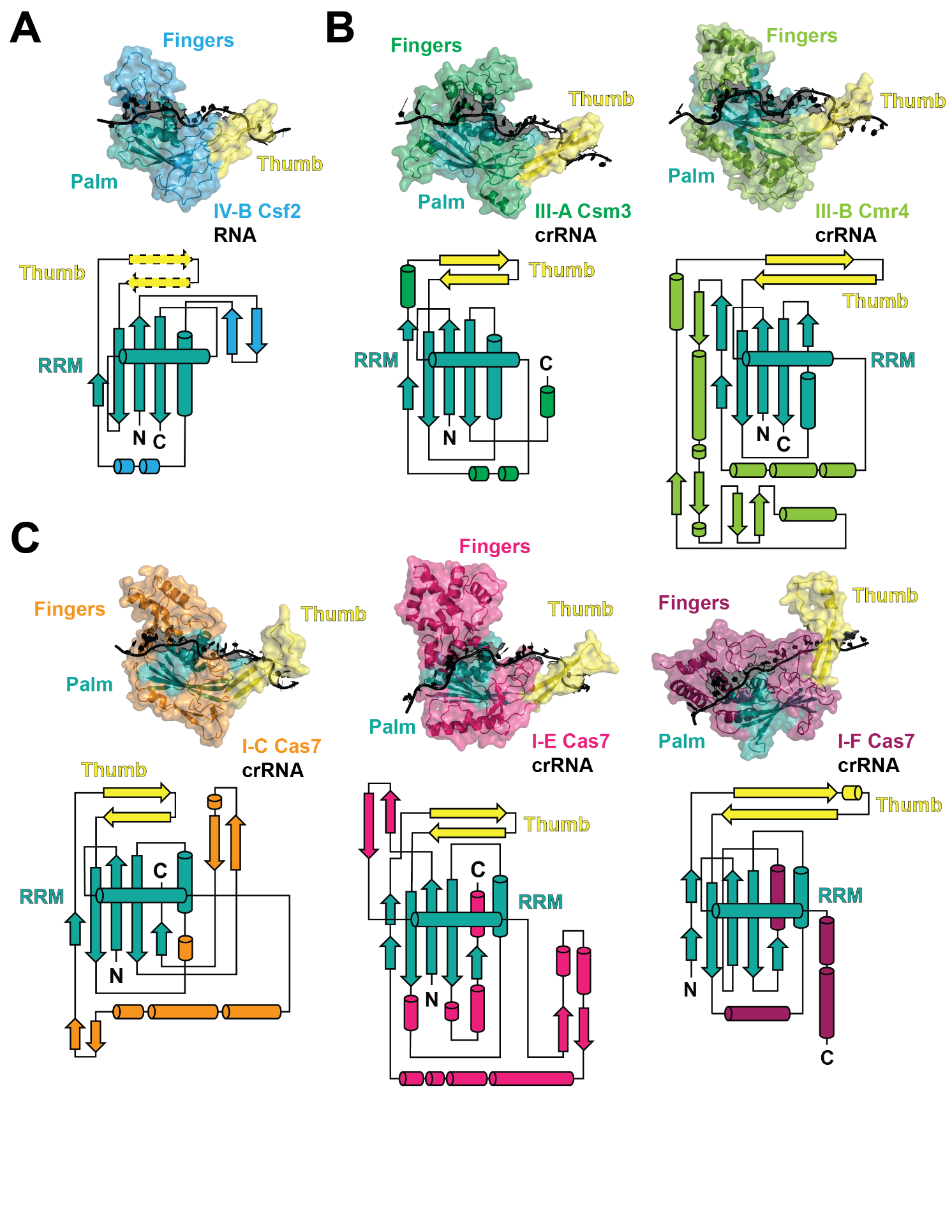
**

**Supplementary Figure 1.** Csf2 is a unique Cas7-like backbone subunit. (**A**) Structure of IV-B Csf2 (PDBid 7JHY) and protein topology map highlighting the hand-like domains typical of Cas7-like proteins. Dashed lines for the thumb indicate that secondary structure is not obvious in the model. (**B**) Structures of III-A Csm3 (PDBid 6O7I) and III-B Cmr4 (PDBid 3X1L) depicted as in (A). (**C**) Structures of Cas7 from I-C (PDBid 7KHA), I-E (PDBid 5H9F), and I-F (PDBid 6B44) depicted as in (A).


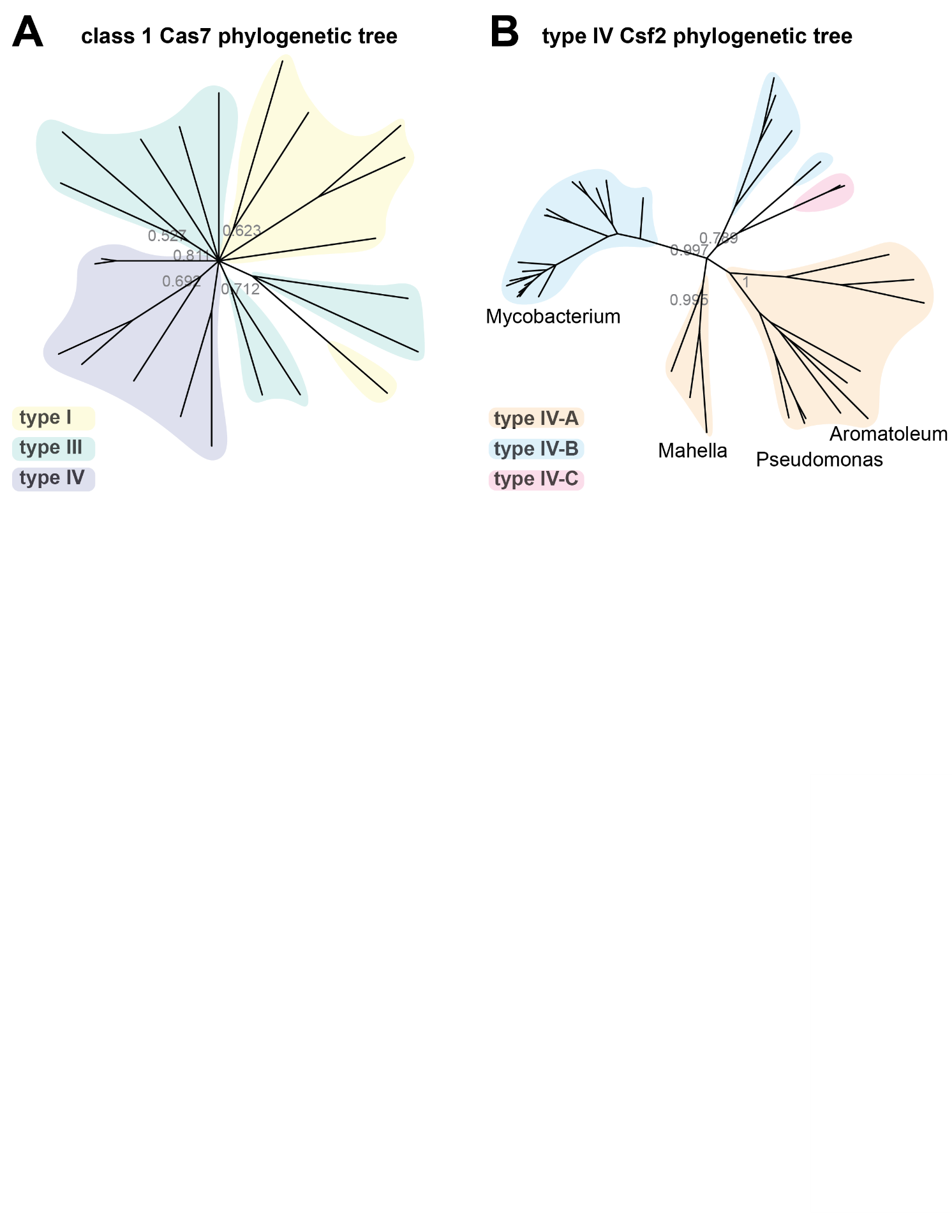


**Supplementary Figure 2.** Csf2 is unique from other Cas7-like subunits and distinct within the type IV subtypes. (**A**) A phylogenetic tree of Csf2 sequences from all three type IV subtypes and a selection of Cas7 sequences from each of the several type I and type III subtypes. (**B**) A phylogenetic tree of Csf2 sequences from all three type IV subtypes. Csf2 sequences from *Mycobacterium JS623* (Zhou et al., 2021), *Mahella australiensis* (Taylor et al., 2019), *Pseudomonas aeruginosa* (Crowley et al., 2019), and *Aromatoleum aromaticum* (Özcan et al., 2018) are indicated. Sequences for both trees were selected from (Makarova et al., 2020).


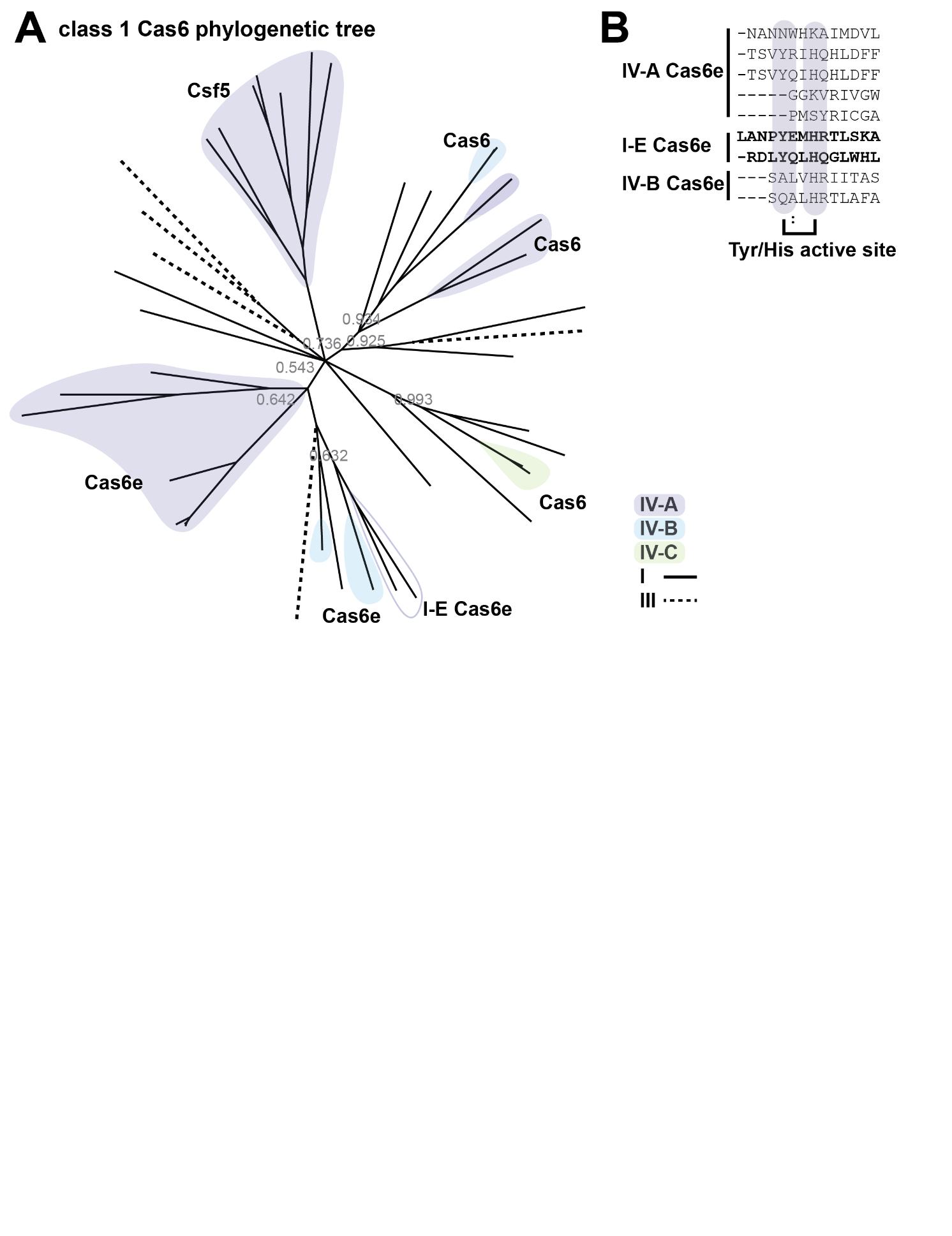


**Supplementary Figure 3.** Type IV Cas6/Csf5 subunits are distinct from Cas6 homologs of other CRISPR systems. (**A**) A phylogenetic tree of type IV Cas6/Csf5 sequences and select type I and type III Cas6 sequences. Sequences were selected from (Makarova et al., 2020). (**B**) A Clustal Omega (Madeira et al., 2019) amino acid sequence alignment of type IV-A and IV-B Cas6e and type I-E Cas6e, highlighting the active site residues. The Cas6e active site is not highly conserved among type IV Cas6e sequences. Bolded sequences have experimentally determined active sites (Jackson et al., 2014; Sashital et al., 2011).


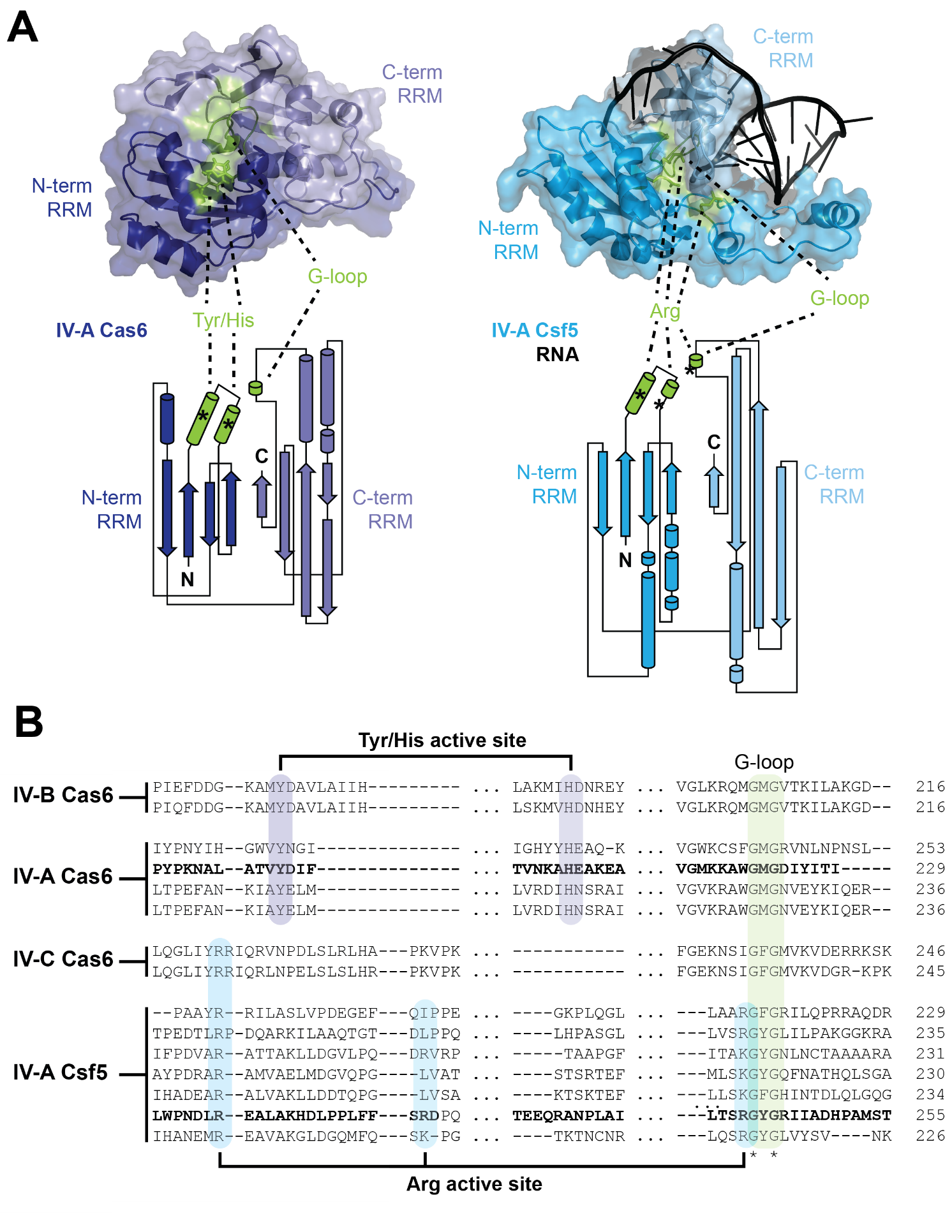


**Supplementary Figure 4.** Type IV Cas6/Csf5 structures and active sites. (**A**) Structural comparisons of apo IV-A Cas6 from Mahella australiensis (PDBid 6NJY) and substrate-bound IV-B Csf5 from Aromatoleum aromaticum (PDBid 6H9I). Both structures are composed of two RRM folds with the active site positioned in the cleft between the folds. (**B**) A Clustal Omega (Madeira et al., 2019) alignment of Csf5 sequences from subtype IV-A and Cas6 sequences from subtypes IV-A, IV-B, and IV-C. The two different active sites architectures (Tyr/His or Arg) are indicated. All aligned sequences share the conserved G-loop motif. The bolded sequences have experimentally confirmed active sites: IV-A Cas6 from *M. australiensis* (Taylor et al., 2019) and IV-A Csf5 from *A. aromaticum* (Özcan et al., 2018).


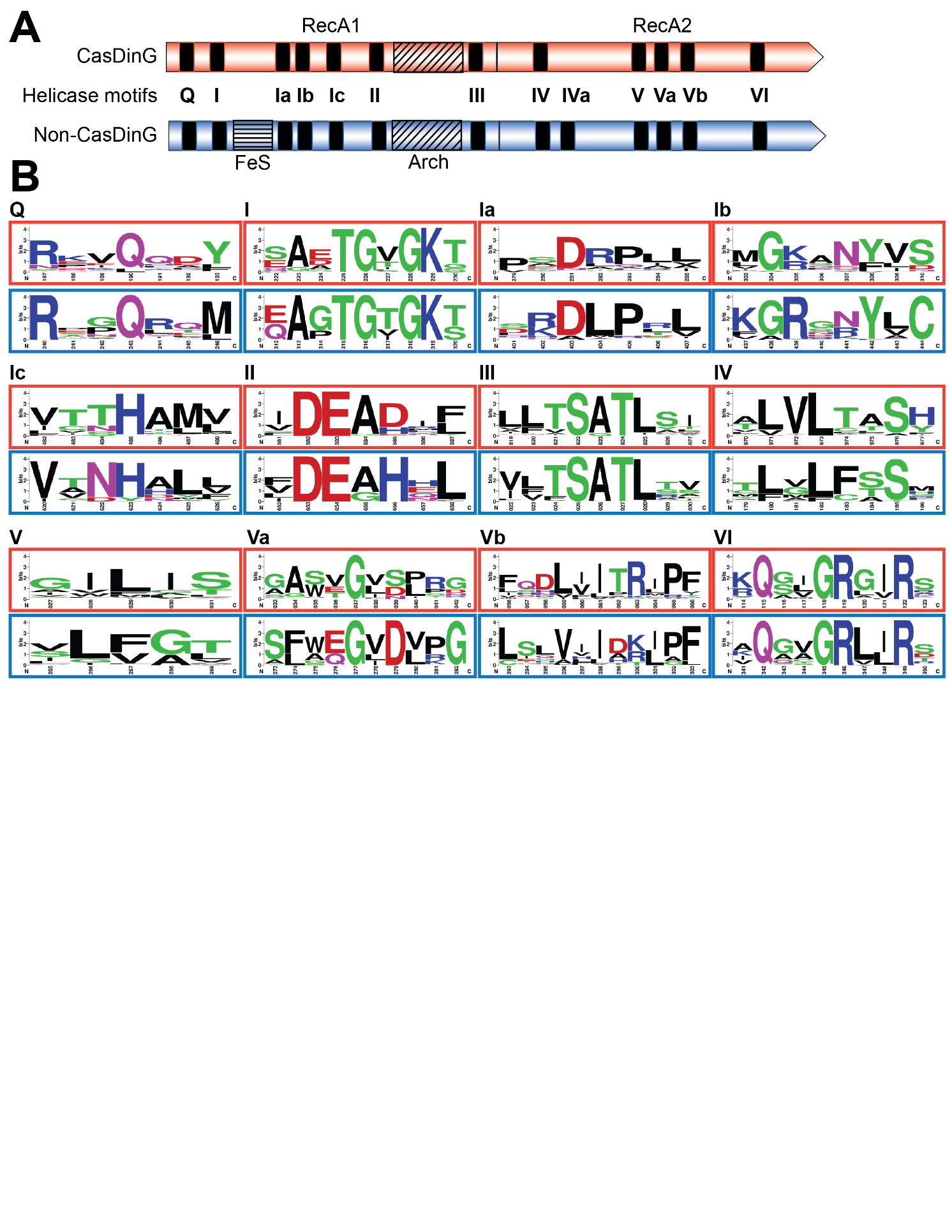


**Supplementary Figure 5.** The conserved helicase motifs of CasDinG and non-CasDinG. (**A**) Cartoon depiction of a Cas- and non-CasDinG primary amino acid sequence indicating the positions of the helicase motifs. (**B**) Weblogos (Crooks, 2004) of the helicase motifs of Cas- and non-CasDinG helicases. CasDinG motifs are outlined in red boxes and non-CasDinG motifs are outlined in blue boxes.


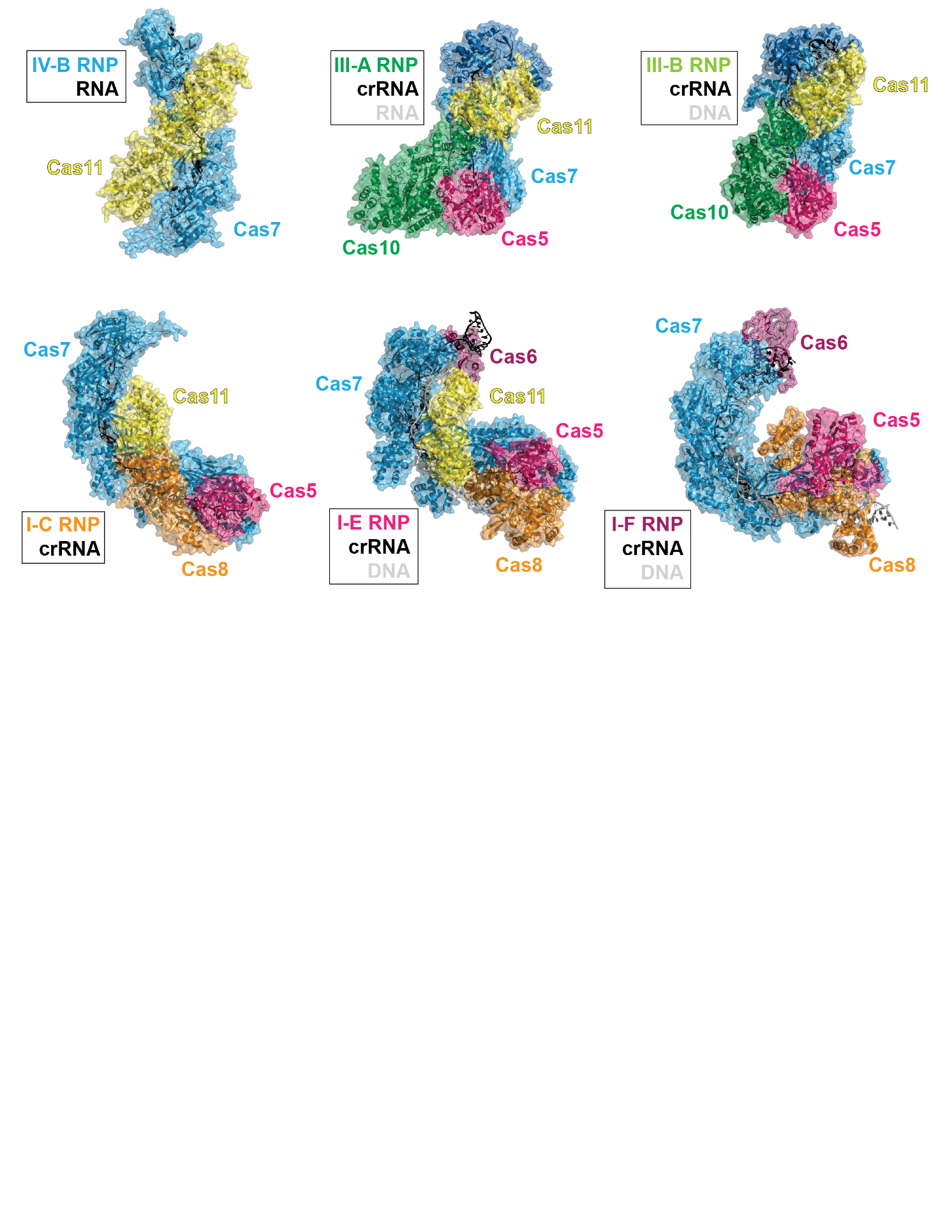


**Supplementary Figure 6.** Structural comparison of class 1 RNP complexes from type IV-B (PDBid 7JHY) (Zhou et al., 2021), type III-A (PDBid 6O7I) (Jia et al., 2019), type III-B (PDBid 3X1L) (Osawa et al., 2015), type I-C (PDBid 7KHA) (O’Brien et al., 2020), type I-E (PDBid 5H9F) (Hayes et al., 2016), and type I-F (PDBid 6B44) (Guo et al., 2017). Complexes are colored by homologous subunits.

# Supplementary Methods

## Sequence alignment and phylogenetic tree generation for DinG and CysH

DinG sequences were identified by searching all bacterial genomes from NCBI (downloaded on 12/03/2020) for homologs of a list of manually curated non-Cas associated dinGs using BLAST (Altschul et al., 1990), as well as matches to HMM profiles for cas-associated dinGs (csf4) using hmmsearch3 (hmmer.org). DinG homologs were classified as cas-associated if they were within 10 genes of a Type IV-A CRISPR-Cas locus. Type IV-A CRISPR-Cas loci were identified by searching for loci which contained matches to HMM profiles for csf2, csf3, csf4, and csf5 within 10 genes of each other. All HMM profiles were obtained from TIGRFAM and Makarova et al., 2020 and searching was performed using hmmsearch3 with an evalue cutoff of 0.001. All BLAST searches were performed with an evalue cutoff of 0.001.

CysH sequences were identified in the same way as dinG sequences. Type IV-B CRISPR-Cas loci were identified by searching for loci which contained homologs of csf2, csf3, and cysH. CysH sequences were considered cas-associated if they were within 15 genes of a Type IV-B CRISPR-Cas locus.

All dinG and cysH sequences were then clustered using MMseqs2 (Steinegger & Söding, 2017) with a minimum sequence identity of 0.80 within a cluster. It was confirmed that no clusters contained both cas and non-cas associated sequences and one representative sequence from each cluster was included in the final tree. Sequences were then aligned with MAFFT (L-INS-i option) and phylogenies were reconstructed using MrBayes (Katoh & Standley, 2013; Ronquist et al., 2012). MrBayes was run until the standard deviation of split frequencies remained below 0.02 for 100000 generations.

## Sequence alignment and phylogenetic tree generation for Cas7 and Cas6 homologs

Alignments were performed and phylogenetic trees generated as described for DinG and CysH. Only sequences identified in Makarova et al., 2020 were included.

# Supplementary material references

Altschul, S. F., Gish, W., Miller, W., Myers, E. W., & Lipman, D. J. (1990). Basic local alignment search tool. *Journal of Molecular Biology*, *215*(3), 403–410. https://doi.org/10.1016/S0022-2836(05)80360-2

Crooks, G. E. (2004). WebLogo: A Sequence Logo Generator. *Genome Research*, *14*(6), 1188–1190. https://doi.org/10.1101/gr.849004

Crowley, V. M., Catching, A., Taylor, H. N., Borges, A. L., Metcalf, J., Bondy-Denomy, J., & Jackson, R. N. (2019). A Type IV-A CRISPR-Cas System in *Pseudomonas aeruginosa* Mediates RNA-Guided Plasmid Interference *In Vivo*. *The CRISPR Journal*, *2*(6), 434–440. https://doi.org/10.1089/crispr.2019.0048

Guo, T. W., Bartesaghi, A., Yang, H., Falconieri, V., Rao, P., Merk, A., Eng, E. T., Raczkowski, A. M., Fox, T., Earl, L. A., Patel, D., & Subramaniam, S. (2017). Cryo-EM Structures Reveal Mechanism and Inhibition of DNA Targeting by a CRISPR-Cas Surveillance Complex. *Cell*, *171*(2), 414-426.e12. https://doi.org/10.1016/j.cell.2017.09.006

Hayes, R. P., Xiao, Y., Ding, F., van Erp, P. B. G., Rajashankar, K., Bailey, S., Wiedenheft, B., & Ke, A. (2016). Structural basis for promiscuous PAM recognition in Type I-E Cascade from E. coli. *Nature*, *530*(7591), 499–503. https://doi.org/10.1038/nature16995

Jackson, R. N., Golden, S. M., Erp, P. B. G. van, Carter, J., Westra, E. R., Brouns, S. J. J., Oost, J. van der, Terwilliger, T. C., Read, R. J., & Wiedenheft, B. (2014). Crystal structure of the CRISPR RNA–guided surveillance complex from Escherichia coli. *Science*, *345*(6203), 1473–1479. https://doi.org/10.1126/science.1256328

Jia, N., Jones, R., Sukenick, G., & Patel, D. J. (2019). Second Messenger cA4 Formation within the Composite Csm1 Palm Pocket of Type III-A CRISPR-Cas Csm Complex and Its Release Path. *Molecular Cell*. https://doi.org/10.1016/j.molcel.2019.06.013

Katoh, K., & Standley, D. M. (2013). MAFFT Multiple Sequence Alignment Software Version 7: Improvements in Performance and Usability. *Molecular Biology and Evolution*, *30*(4), 772–780. https://doi.org/10.1093/molbev/mst010

Madeira, F., Park, Y. mi, Lee, J., Buso, N., Gur, T., Madhusoodanan, N., Basutkar, P., Tivey, A. R. N., Potter, S. C., Finn, R. D., & Lopez, R. (2019). The EMBL-EBI search and sequence analysis tools APIs in 2019. *Nucleic Acids Research*, *47*(W1), W636–W641. https://doi.org/10.1093/nar/gkz268

Makarova, K. S., Wolf, Y. I., Iranzo, J., Shmakov, S. A., Alkhnbashi, O. S., Brouns, S. J. J., Charpentier, E., Cheng, D., Haft, D. H., Horvath, P., Moineau, S., Mojica, F. J. M., Scott, D., Shah, S. A., Siksnys, V., Terns, M. P., Venclovas, Č., White, M. F., Yakunin, A. F., … Koonin, E. V. (2020). Evolutionary classification of CRISPR–Cas systems: A burst of class 2 and derived variants. *Nature Reviews Microbiology*. https://doi.org/10.1038/s41579-019-0299-x

O’Brien, R. E., Santos, I. C., Wrapp, D., Bravo, J. P. K., Schwartz, E. A., Brodbelt, J. S., & Taylor, D. W. (2020). Structural basis for assembly of non-canonical small subunits into type I-C Cascade. *Nature Communications*, *11*. https://doi.org/10.1038/s41467-020-19785-8

Osawa, T., Inanaga, H., Sato, C., & Numata, T. (2015). Crystal Structure of the CRISPR-Cas RNA Silencing Cmr Complex Bound to a Target Analog. *Molecular Cell*, *58*(3), 418–430. https://doi.org/10.1016/j.molcel.2015.03.018

Özcan, A., Pausch, P., Linden, A., Wulf, A., Schühle, K., Heider, J., Urlaub, H., Heimerl, T., Bange, G., & Randau, L. (2018). Type IV CRISPR RNA processing and effector complex formation in Aromatoleum aromaticum. *Nature Microbiology*. https://doi.org/10.1038/s41564-018-0274-8

Ronquist, F., Teslenko, M., van der Mark, P., Ayres, D. L., Darling, A., Höhna, S., Larget, B., Liu, L., Suchard, M. A., & Huelsenbeck, J. P. (2012). MrBayes 3.2: Efficient Bayesian Phylogenetic Inference and Model Choice Across a Large Model Space. *Systematic Biology*, *61*(3), 539–542. https://doi.org/10.1093/sysbio/sys029

Sashital, D. G., Jinek, M., & Doudna, J. A. (2011). An RNA-induced conformational change required for CRISPR RNA cleavage by the endoribonuclease Cse3. *Nature Structural & Molecular Biology*, *18*(6), 680–687. https://doi.org/10.1038/nsmb.2043

Steinegger, M., & Söding, J. (2017). MMseqs2 enables sensitive protein sequence searching for the analysis of massive data sets. *Nature Biotechnology*, *35*(11), 1026–1028. https://doi.org/10.1038/nbt.3988

Taylor, H. N., Warner, E. E., Armbrust, M. J., Crowley, V. M., Olsen, K. J., & Jackson, R. N. (2019). Structural basis of Type IV CRISPR RNA biogenesis by a Cas6 endoribonuclease. *RNA Biology*, 1–10. https://doi.org/10.1080/15476286.2019.1634965

Zhou, Y., Bravo, J. P. K., Taylor, H. N., Steens, J. A., Jackson, R. N., Staals, R. H. J., & Taylor, D. W. (2021). Structure of a type IV CRISPR-Cas ribonucleoprotein complex. *IScience*, 102201. https://doi.org/10.1016/j.isci.2021.102201
